# Psychological Aspects and Depression in Patients with Symptomatic Keratoconus

**DOI:** 10.1155/2018/7314308

**Published:** 2018-05-29

**Authors:** Marilita M. Moschos, Nikolaos S. Gouliopoulos, Chris Kalogeropoulos, Sofia Androudi, George Kitsos, Dimitrios Ladas, Michael Tsatsos, Irini Chatziralli

**Affiliations:** ^1^1st Department of Ophthalmology, Medical School of University of Athens, Athens, Greece; ^2^Department of Ophthalmology, Medical School of University of Ioannina, Ioannina, Greece; ^3^Department of Ophthalmology, Medical School of University of Larisa, Larisa, Greece; ^4^Royal Eye Infirmary, Dorset County Hospital, Dorchester, UK

## Abstract

**Purpose:**

To assess the psychological status of keratoconus sufferers and to determine the relationship between depression and visual impairment in this group of patients.

**Methods:**

Fifty-six patients with keratoconus and forty-seven age- and gender-matched healthy control subjects were retroprospectively analyzed. Every participant underwent a complete ophthalmological examination. Keratoconus diagnosis was confirmed with corneal topography and tomography. Zung Depression Inventory Questionnaire and Patient Health Questionnaire-9 (PHQ-9) were completed by everyone.

**Results:**

Visual acuity (logMAR 0.53 ±0.30 versus 0.11 ± 0.16), PHQ-9 score (10.20 ± 4.00 versus 5.40 ± 5.01), and Zung score (46.52 ± 8.70 versus 38.53 ± 8.41) showed a statistically significant difference between keratoconus patients and healthy controls (*p* < 0.001 for all). Worse visual acuity was strongly correlated with older individuals (rho = 0.339, *p*=0.011) and higher PHQ-9 (rho = 0.765, *p* < 0.001) and Zung score (rho = 0.672, *p* < 0.001).

**Conclusion:**

Depressive disorders appear to be directly associated with keratoconus, both in frequency and intensity. Worse visual acuity and older age could be identified as predictive factors for their emotional status. Moreover, the disease itself could be recognized as an independent risk factor for depression development, underlying the need for close monitoring and supportive management. To the best of our knowledge, our study is the first in the literature to elaborate the association between keratoconus and depression, by assessing two different questionnaires simultaneously.

## 1. Introduction

Keratoconus (KC) is a noninflammatory, progressive, and bilateral but usually asymmetric corneal disorder, characterized by stromal thinning, iron deposition in the epithelial basement membrane, breaks in Bowman's layer, steepening of central cornea, and in more advanced stages by corneal ectasia or even acute stromal oedema (hydrops) [1–3]. Its exact etiology and pathogenesis remain unknown. Its prevalence is reported to range from 0.3 to 2,300 per 1,00,000 in Russia and central India, respectively [1–3].

In the early stages, symptomatology is usually vague, with blurred vision, due to changes in refractive error and photosensitivity, being the major patients' complains, thus needing corneal topography to confirm the diagnosis [2]. In more advanced stages, severe visual impairment is present due to irregular astigmatism, myopia, corneal scarring, and hydrops.

Depression is a growing public health disorder worldwide, affecting especially the elderly and patients with chronic health problems, such as cancer, chronic heart disease, and chronic obstructive pulmonary disease [4–7]. Depression is associated with increased morbidity and mortality, or suicidal attempts [8]. Although the disease could be easily identified and effectively treated in early stages [9, 10], it usually goes undetected and insufficiently treated for long periods of time [11–13]. In primary care, numerous questionnaires have been used for screening depression, including among others Geriatric Depression Scale [14], Hospital Anxiety and Depression Scale [15], 9-item Patient Health Questionnaire (PHQ-9) [16], Zung Depression Inventory-Self-Rating Depression Scale (Zung SDS) [17], Beck Depression Inventory [18], Hamilton Rating Scale for Depression [19], 12- or 36-item Short Form Health Survey [20], and the International Classification of Diseases, Ninth Revision, Clinical Modification (ICD-9-CM) codes [21].

Visual impairment is a result by numerous disorders, affecting crucially patients' quality of life, and is regarded as one of the most common chronic causes of psychological distress, particularly in the elderly [22]. It is well established that self-reported decline in visual function is accompanied by depressive symptoms [23, 24], whereas the association between visual acuity and depression remains controversial [23–27].

Several studies have evaluated the aforementioned association, detecting depression in patients with age-related macular degeneration [25], glaucoma [28], retinitis pigmentosa [29], and Stargardt disease [30]. As for the KC, studies demonstrated controversial conclusions. Gorskova et al. identified a positive correlation only in women [31], while Woodward et al. rejected the existence of such an association [32].

As early detection and appropriate treatment of depression could inhibit effectively its progression to more severe stages, the purpose of our study was to examine the association of KC and depression, using for first time both PHQ-9 and Zung Depression Inventory (Zung SDS) questionnaires, and to identify whether worse objective vision in KC patients, expressed by visual acuity, is associated with the severity of depression.

## 2. Materials and Methods

In this case control study, we included 56 KC patients (61% male) and 47 (64% male) age- and gender-matched healthy control subjects (CL). The KC subjects were recruited from the Laboratory of Electrophysiology, 1st Department of Ophthalmology, University of Athens, Greece.

The CL population consisted of subjects that referred to the outpatient department of ophthalmology for ocular examination and who had no systemic or ocular disorders. All the individuals were Caucasian, middle aged, and nonsmokers. Furthermore, none of them suffered from diabetes mellitus, hypertension, glaucoma, cataract, or any other ocular disorder that could contribute to visual deterioration.

The study was performed according to the Helsinki Declaration and was approved by the ethical committee of our hospital, General Hospital of Athens “G. Gennimatas.” Written informed consent was obtained by everyone that took part in the study.

All the participants were examined thoroughly, clinically, and topographically and completed the PHQ-9 and Zung SDS questionnaires, translated and validated in Greek. Corrected distance visual acuity (CDVA) was measured with Snellen charts (measured in decimals). For KC patients their usual best correction (spectacles or contact lenses) was utilized. The values were converted in a logarithm of the minimum angle of resolution (logMAR) scale for statistical purposes, and mean CDVA score was used in the statistical analysis. Slit-lamp biomicroscopy examined the existence of stromal corneal thinning, Vogt striae, or a Fleischer ring. Retinoscopy after pupil dilation (20 minutes after phenylephrine 2.5% and cyclopentolate 1% drops had been instilled in the eye) determined the presence or absence of retroillumination signs of KC, such as the oil droplet sign and scissoring of the red reflex.

The criteria used in KC diagnosis included (1) distortion of the corneal surface; (2) visual acuity reduction; (3) stromal thinning within central cornea, using a comprehensive ophthalmic examination including, visual acuity measurement, and corneal imaging; (4) presence of Vogt striae; and (5) presence of cone upon Scheimpflug topography with irregular astigmatism. In order to assign a subject to KC group, it was needed both the presence of at least one clinical sign of KC and a confirmatory videokeratographic map, with an asymmetric bowtie with skewed radial axis above and below the horizontal meridian (AB/SRAX) pattern [1].

Zung SDS questionnaire is a short self-rating scale regarding the affective, psychological, and somatic symptoms of depression [17, 33]. It consists of 20 questions which address the most commonly found diagnostic criteria of depression during patients' interviews. Ten questions are worded positively and the rest negatively. Each one is scored on a scale of 1–4 (a little of the time to most of the time) according to the frequency of each feeling during the preceding week. Total scores range from 20 to 80. Scores below 49 are regarded normal, whereas 50–59 exhibit mild, 60–69 moderate, and over 70 severe depression [34–36].

PHQ-9 depression scale is a questionnaire used for screening, identification, and evaluation of depression, with well-established validity and reliability [16, 37–39]. It assesses patient's emotional status over the past two weeks, by scoring each of the 9 criteria of the Diagnostic and Statistical Manual of Mental Disorders- IV (DSM-IV) [40]. The responses are rated from “0” (not at all) to “3” (nearly every day). Total score is the sum of all the answers, ranging from 0 to 27, with elevated values indicating more severe depressive symptoms. Scores less than 5 demonstrate the absence of a depressive disorder, 5 to 9 exhibit mild, 10 to 14 moderate, and over 15 severe depression [16].

Statistical comparisons between patients and controls were performed using Mann–Whitney–Wilcoxon (MWW) test, after confirmation of nonnormality with a Kolmogorov–Smirnov test. The correlations of the Zung SDS score and the PHQ-9 score to CDVA, age, and sex were analyzed by the Spearman correlation test. Statistical analysis was performed using the SPSS 24.0 software (IBM SPSS Inc., Chicago, IL, USA). A *p* value less than 0.05 was considered statistically significant.

## 3. Results

The demographic and clinical data of the participants are summarized in Table 1. The KC group consisted of 56 subjects, whereas the CL group comprised 47 participants. No significant differences existed in mean age (41 ± 7 years versus 42 ± 9 years, resp.) and gender status (61% male versus 64% male, resp.) between the groups.

Subjects with KC, compared to CL subjects, had significantly elevated both PHQ-9 score (10.20 ± 4.00 versus 5.40 ± 5.01, *p* < 0.001) and Zung SDS score (46.52 ± 8.70 versus 38.53 ± 8.41, *p* < 0.001). Moreover, CDVA in KC patients was significantly worse compared to CL (CDVA logMAR 0.53 ± 0.30 versus 0.11 ± 0.16, *p* < 0.001).

According to PHQ-9 score, 12.5% of KC patients did not suffer from depression, while 46.4% encountered mild, 28.6% moderate, and 12.5% severe depressive symptoms. Zung SDS score detected no depressive symptomatology in 58.9% of the KC patients, mild symptomatology in 33.9%, and moderate in 7.2% of them.

In Table 2 are presented the intercorrelations among the examined parameters in the KC group. Older participants had worse CDVA (rho = +0.339, *p*=0.011) and elevated PHQ-9 score and Zung SDS score (rho = +0.444, *p*=0.001 and rho = +0.422, *p*=0.001, resp.). No significant correlations were associated with age. Patients with worse CDVA presented significantly higher PHQ-9 score and Zung SDS score (*p* < 0.001 for both). Finally, PHQ-9 score was positively and strongly correlated with Zung SDS score (rho = 0.907, *p* < 0.001).

## 4. Discussion

In the present study, we demonstrated that depression occurs more frequently and with greater intensity in KC patients compared to age and gender-matched healthy control subjects. The possible association between KC and depression had not been thoroughly investigated. Although the hypothesis that KC is accompanied by depression has been rejected by Woodward et al. [32], this association appeared to demonstrate a gender bias [31]. Cingu et al. reported significant improvement of anxiety after cross-linking (CXL) in KC patients; however, this intervention did not appear to influence the depressive status of KC patients [41]. To the best of our knowledge, our study is the first to report a significant association between depression and KC, regardless of the participants' age and gender, by assessing two different questionnaires simultaneously.

Low visual acuity attributed to conditions, such as age-related macular degeneration (ARMD) [26] and retinitis pigmentosa [29], has been associated been associated with depression. We demonstrated that worse objective visual function, expressed by lower CDVA, is mildly (0.339) but significantly (*p* < 0.05) correlated with older age and with more intense depressive symptoms. Previous studies using solely the PHQ-9 questionnaire suggested self-reported visual deterioration, rather than lower best corrected visual acuity (BCVA) correlation with depression [23, 24]. The differences could be at least partly attributed to the fact that Morse [23], and Zhang et al. [24], examined the association of visual impairment and depression in a population of US citizens, regardless of the presence or not of an ocular disorder and without taking into account the causes of vision loss. The KC participants in our study did not suffer from other common causes of depression, and therefore our findings suggest that not only visual impairment, but also the disease itself plays a key role in the development of depression.

Interestingly, we found that PHQ-9 score and Zung SDS score were positively and strongly intercorrelated (rho = +0.907, *p* < 0.001) in the KC population. Although PHQ-9 usefulness in assessing and grading depression in visually impaired people is well established [42], Zung SDS appears to be a valuable tool in screening depression in adults [17], while its usefulness in grading depression severity remains controversial [43]. In our study, both questionnaires showed consistency and sensitivity in detecting depressive symptoms in KC patients.

Although depression can be readily diagnosed and treated, especially in early stages, it can easily remain undetected and therefore untreated for prolonged periods [9, 10, 44]. Eye care professionals should be aware of the increased prevalence of depression in patients with visual impairment, especially with growing age. Screening tools, such as PHQ-9 and Zung SDS questionnaires, could help ophthalmologists detect depressive symptoms even in early stages and thus urge prompt referral for psychiatric evaluation and possible treatment. It is important to note that in visually impaired patients, depression may cause poorer adherence to treatment, which in turn accelerates the progression of irreversible vision loss, resulting in a more pronounced depressive status [45, 46].

Our study included KC patients with measured CDVA, effectively detected depression using short, easily administered, and well-validated tools such as PHQ-9 and Zung SDS, and it was the first to underline the role of KC itself in the development of depression. Possible limitation of our study is the fact that the KC subjects were in different disease stages which impedes the assessment of disease duration on presence and severity of depression.

## 5. Conclusions

Keratoconus patients encounter more often depressive symptoms compared to healthy subjects without visual impairment. The frequency and the severity of the symptoms correlate with patients' age and CDVA, suggesting that worse vision and older age (at presentation) in KC patients could be identified as predictive factors of their emotional status. Finally, our study demonstrates the usefulness of PHQ-9 and Zung SDS questionnaires as a screening tool for depression in KC patients and underlines the need for close monitoring and supportive management of this vulnerable population.

## Figures and Tables

**Table 1:** Demographic and clinical characteristics of patients with keratoconus and controls.

	Patients (*n*=56)	Controls (*n*=47)	*p* value
Age (years)	41 ± 7	42 ± 9	0.65
Gender, male (%)	61	64	0.84
CDVA logMAR	0.53 ± 0.30	0.11 ± 0.16	**<0.001**
PHQ-9 score	10.20 ± 4.00	5.40 ± 5.01	**<0.001**
Zung score	46.52 ± 8.70	38.53 ± 8.41	**<0.001**

**Table 2:** Intercorrelations among the examined parameters in patients with keratoconus.

	Age	Sex	CDVALogMAR	PHQ-9
Sex	0.01 (*p*=0.941)			
CDVA logMAR	0.339 (*p*=0.011)^*∗*^	−0.151 (*p*=0.267)		
PHQ-9 score	0.444 (*p*=0.001)^*∗∗*^	0.224 (*p*=0.114)	0.765 (*p* < 0.001)^*∗∗*^	
Zung score	0.422 (*p*=0.001)^*∗∗*^	0.283 (*p*=0.102)	0.672 (*p* < 0.001)^*∗∗*^	0.907 (*p* < 0.001)^*∗∗*^

^*∗*^ *p* < 0.05; ^*∗∗*^*p* < 0.001.
